# Supplementary figures and images for: Variations in de novo donor-specific antibody development among HLA-DQ mismatches in kidney transplant recipients
Source: PLoS One. 2025 Apr 15;20(4):e0321629. doi: 10.1371/journal.pone.0321629 (PMC11999115; doi:10.1371/journal.pone.0321629)

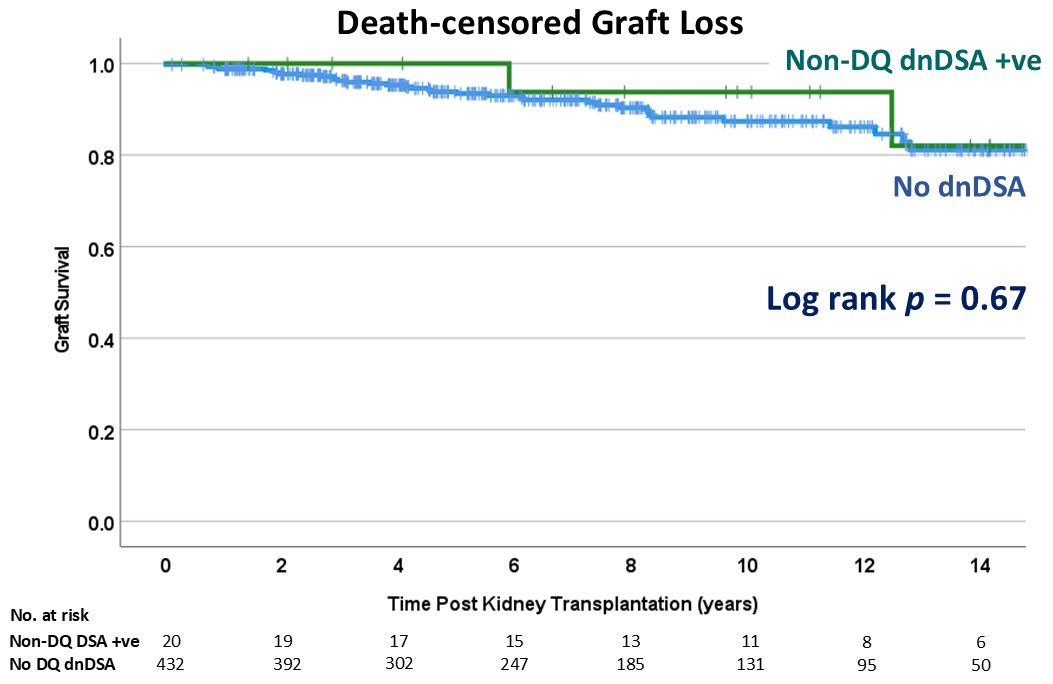

Supplement: S1 Table — (DOCX) [file pone.0321629.s001.tif]

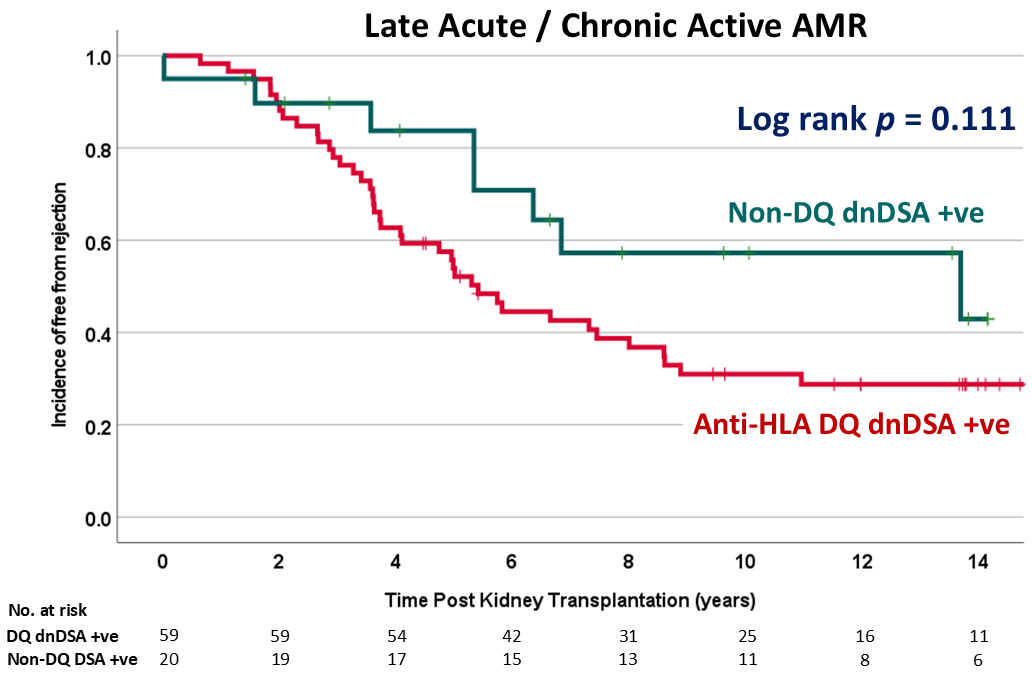

Supplement: S1 Fig — (TIF) [file pone.0321629.s002.tif]

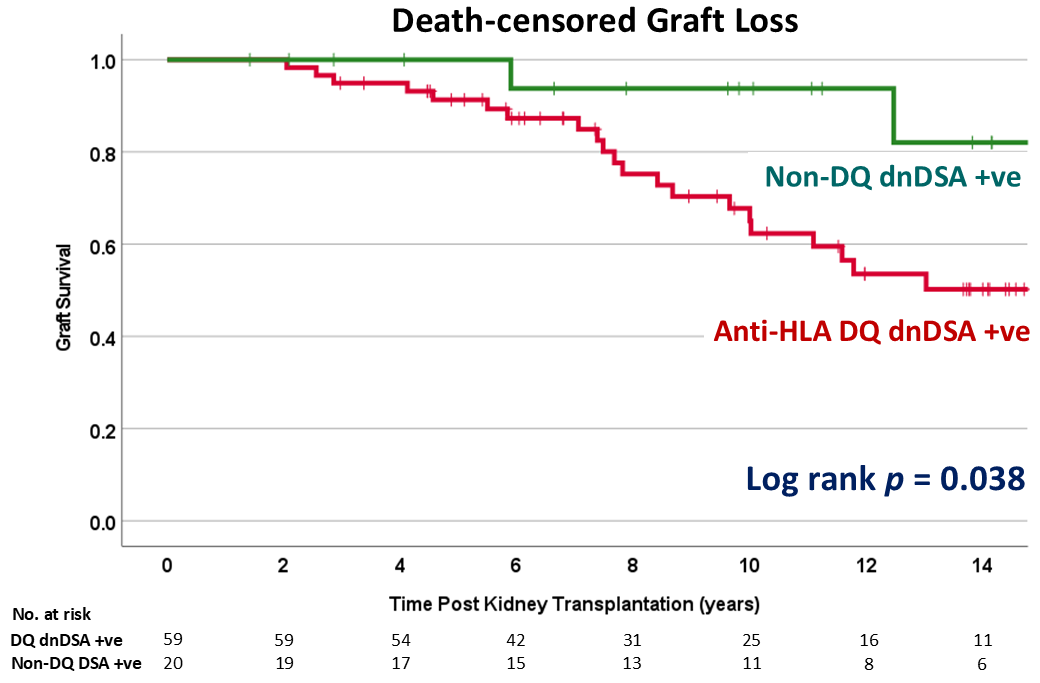

Supplement: S2 Fig — (TIF) [file pone.0321629.s003.tif]
