# Supplementary material for: Variations in de novo donor-specific antibody development among HLA-DQ mismatches in kidney transplant recipients
Source: PLoS One. 2025 Apr 15;20(4):e0321629. doi: 10.1371/journal.pone.0321629 (PMC11999115; doi:10.1371/journal.pone.0321629)
Supplement: Attachment — Submitted filename: Responses to reviewers.docx [file pone.0321629.s006.docx]

Division of Nephrology

Department of Medicine

Faculty of Medicine Siriraj Hospital, Mahidol University

2 Prannok Road, Bangkoknoi

Bangkok 10700, Thailand

10 January 2025

Dear Editorial Board of *Plos One* Journal,

We appreciate the time you have taken to review our manuscript. It is with great pleasure that we note the consideration of our submission (Submission ID: PONE-D-24-21041), titled **“Variations in De Novo Donor-Specific Antibody Development Among HLA-DQ Mismatches in Kidney Transplant Recipients”** for potential publication by the Editorial Board of *Plos One*. We extend our gratitude to the reviewers for their invaluable comments. We have thoroughly revised our manuscript in response to these comments, and our responses to the reviewers' comments are provided below.

Thank you once again for considering our manuscript for publication. We anticipate a favorable decision and remain available to address any further inquiries or concerns you may have.

Best regards,

Nuttasith Larpparisuth, MD

Associate Professor of Medicine

Faculty of Medicine Siriraj Hospital, Mahidol University

2 Wanglang Road, Bangkoknoi

Bangkok 10700, Thailand

Tel: (+66) 2-419-8383

E-mail: Nuttasith.lap@gmail.com

**Journal Requirements**

In the online submission form, you indicated that all relevant data are within the manuscript. The datasets that support the findings of this study are available from the corresponding author upon reasonable request.

All PLOS journals now require all data underlying the findings described in their manuscript to be freely available to other researchers, either 1. In a public repository, 2. Within the manuscript itself, or 3. Uploaded as supplementary information.

This policy applies to all data except where public deposition would breach compliance with the protocol approved by your research ethics board. If your data cannot be made publicly available for ethical or legal reasons (e.g., public availability would compromise patient privacy), please explain your reasons on resubmission and your exemption request will be escalated for approval.

**Responses**:

We agree with journal requirement. We have already attached our dataset regarding this study in this manuscript, as your recommendations. For rebuttal, we have already addressed all comments from reviewers with a point-by-point response, as recommended in the guidelines.

**From Reviewer #1,**

This is a single-centre, retrospective study conducted on a cohort of kidney transplant and SPK first-graft recipients over a 14yrs period aimed to identify the specific HLA-DQB1 mismatches associated with de novo DSA occurrence and their impact on graft survival. Authors found that patients with HLA-DQ7 and HLA-DQ9 MMs had the highest risk of developing de novo DSAs, and that DSAs against DQB1 MM were associated with detrimental graft outcomes.

It is interesting to see that authors have interestingly managed to cast some light on the importance of matching against DQB1 antigens, raising concern about the higher risk of developing DSAs against DQ7 and DQ9. The association between DSAs against DQB1 antigens and rejection / allograft loss is not a new finding and has extensively been described in the literature (of note - the works from Aleksandar Senev and co-workers should be cited as remarkable studies highlighting the role of DSAs against DQs and poor transplant outcomes).

**Responses:**

We sincerely thank the reviewer for the thoughtful and constructive feedback on our manuscript. We agree that the association between DSAs against HLA-DQB1 mismatches and adverse transplant outcomes has been previously described in the literature. However, we believe our study provides additional insights by highlighting the specific risk associated with HLA-DQ7 and HLA-DQ9 mismatches, which has not been extensively explored.

We appreciate your suggestion to cite the important work of Senev A et al., which has significantly contributed to the understanding of HLA mismatches and their impact on transplant outcomes. We have now cited the study by Senev A et al. in our manuscript:

Senev A, Coemans M, Lerut E, Van Sandt V, Kerkhofs J, Daniëls L, et al. Eplet Mismatch Load and De Novo Occurrence of Donor-Specific Anti-HLA Antibodies, Rejection, and Graft Failure after Kidney Transplantation: An Observational Cohort Study. J Am Soc Nephrol. 2020;31(9):2193-2204.

This citation has been added as reference number 5 to support the significance of HLA-DQ mismatches in the development of dnDSAs and their impact on transplant outcomes.

1. Is the survival analysis the result of a crude analysis? Although well known from the literature, it is difficult to state that DSAs against DQB1 MMs are associated with detrimental outcomes in the in-study cohort if the survival analysis doesn't consist in a cox-regression taking into account
1) HLA-A / HLA-B MM (and possibly HLA-Cw where available)

2) Co-existence of class I and class II DSAs

3) Prevalence of cellular rejection and BK nephropathy

**Responses**

Thank you for your thoughtful comment regarding our survival analysis. We initially reported crude survival analysis in the manuscript, showing that dnDSAs to HLA-DQ were significantly associated with inferior long-term graft survival. In response to your suggestion, we have now performed a Cox proportional hazards regression model to account for potential confounding factors, including HLA-A, HLA-B mismatches, the presence of class I and class II DSAs, and acute cellular rejection.

Our adjusted analysis confirmed that dnDSAs to HLA-DQ were significantly associated with overall graft failure (HR: 3.875, 95% CI: 2.12–7.07). These findings strengthen the association between dnDSAs to HLA-DQ and detrimental graft outcomes in our cohort.

Updated text in manuscript (Results Section):

"While the 5-year death-censored graft survival was similar for recipients with and without dnDSAs (94.6% vs. 93.8%, P = 0.452), the 10-year survival was significantly lower for recipients with dnDSAs to HLA-DQ compared to those without dnDSAs (70.2% vs. 87.8%; P = 0.001). Overall graft survival was also inferior in recipients with dnDSAs to HLA-DQ (log-rank P < 0.001; Fig. 4B). In an adjusted Cox proportional hazards model, the presence of dnDSAs to HLA-DQ remained significantly associated with overall graft failure (HR: 3.875, 95% CI: 2.12–7.07) (Table 3)."

We acknowledge that we have limited data on BK nephropathy in our cohort, and therefore this factor was not included in the Cox regression model. We have added this as a limitation in the revised manuscript.

Updated text in manuscript (Discussion Section):

"Incomplete data on BK nephropathy in our cohort, prevented us from including this factor in the survival analysis. BK nephropathy is a known contributor to graft loss, and future studies with complete data on this variable would provide a more comprehensive understanding of the impact of dnDSA on graft outcomes. "

Thank you for your valuable suggestion, which has helped improve the depth and rigor of our analysis. We believe that the detrimental outcomes associated with anti-DQ antibodies observed in our study are due to the long follow-up period.

2. Was the incidence of AMR under-estimated? according to the methods, a biopsy was done only if DSA MFI > 5,000 or change in function - early AMR stage may have been missed.

**Responses**

We appreciate your insightful comment regarding the potential underestimation of antibody-mediated rejection (AMR). We acknowledge that early AMR could have been missed, particularly in cases where donor-specific antibodies (DSAs) had a mean fluorescence intensity (MFI) below the threshold for performing a biopsy. In response to your suggestion, we have revised the limitations section of our manuscript to reflect this concern. The statement now reads:

“Second, protocol biopsy was not performed routinely, and graft biopsy was not performed in all cases with dnDSAs, as some cases resolved after adjusting immunosuppression. Therefore, early AMR may have been underdiagnosed, particularly in cases with low DSA MFI.”

This revision highlights the possibility of missed early AMR cases and addresses the limitations in our study design. We believe this addition will improve the clarity and transparency of our manuscript.

3. Did the authors look into the selective pathogenicity of DSAs against specific DQs?

**Responses**

We appreciate your perceptive comment. We have considered the selective pathogenicity of DSAs against specific HLA-DQ antigens, particularly the higher risk associated with HLA-DQ7 and HLA-DQ9 mismatches. Interestingly, a previous study in heart transplantation also reported an association between HLA-DQ7 and de novo DSA development, which supports our findings.

As discussed in our manuscript, we analyzed the epitope load for each HLA-DQ mismatch and found that the epitope load for DQ7 and DQ9 is lower than that of other DQ types, such as DQ6, which exhibited a lower incidence of DSA formation. This suggests that the risk of DSA development may not be fully explained by the quantitative epitope mismatch load alone.

Therefore, we hypothesize that certain epitopes may have higher immunogenic potential despite a lower overall mismatch load. We have included this point in the Discussion section: “Further studies are needed to explore the qualitative differences in epitope immunogenicity, as some epitopes may elicit stronger alloimmune responses than others.”

4. Minor revision: page 7 line 112: the term 'repeated DQ MM' is inappropriately used in my opinion, as it should instead refer to MMs which recurs in recipients of >1 graft.

**Responses**:

We appreciate your insightful comment regarding the term "repeated DQ MM." We agree that, in transplant immunology, this term typically refers to mismatches that recur in recipients of multiple grafts. However, in our manuscript, "repeated DQ MM" was used to describe cases where both donor HLA-DQ antigens mismatched the recipient with the same DQ allele (e.g., both donor HLA-DQ antigens are DQ5). To avoid confusion, we will revise the term to "duplicate DQ MM" to more accurately reflect the intended meaning and differentiate it from the standard usage of "repeated DQ MM" in the context of multiple grafts. We will update the manuscript accordingly for clarity.

**From Reviewer #2,**

In their manuscript "Variations in De Novo Donor-Specific Antibody Development Among HLA-DQ Mismatches in Kidney Transplant Recipients" Skulratanasak and colleagues retrospectively investigated a cohort of 491 kidney transplant recipients with HLA-DQB1 mismatches. They analyzed individually DQ loci carried by the recipient, and the likelihood of developing donor specific antibodies (DSAs). They found that patients who developed DSAs to HLA-DQ had increased risk of rejection and graft loss, in line with previously published results. They found that some recipient antigen mismatches such as DQ7 and DQ9 are at highest risk of inducing DSAs. The methodology used is mostly appropriate. The manuscript is sometimes difficult to follow. While there is some originality to the main finding of the manuscript, its clinical relevance needs to be further clarified. Specifically, it remains to be demonstrated whether the stratification in risk of developing DSAs (figure 3) also correlates with stratified rejection-free survivals and graft outcomes. I would encourage the authors to consider the following additional issues should they consider resubmitting the manuscript to a different journal.

**Responses:**

We sincerely thank the reviewer for the thorough evaluation of our manuscript and for providing constructive feedback. We appreciate the recognition of the originality of our study in identifying specific HLA-DQ mismatches, particularly DQ7 and DQ9, as high-risk for dnDSA development and the association with graft outcomes.

We greatly appreciate the reviewer’s encouragement to improve the clarity of our manuscript and will take this opportunity to strengthen both the scientific content and clinical implications of our findings. We hope that the revisions made in response to the reviewer’s suggestions will provide more clarity on the clinical relevance of our study.

1. Data on DR mismatches should be provided. In its current form, it is impossible to determine whether clinical outcomes were influenced by DR mismatches

**Responses:**

Thank you for your insightful comment regarding the potential influence of HLA-DR mismatches on clinical outcomes. We have now included data on HLA-DR mismatches in Table 2 of the revised manuscript to provide additional context.

In our Cox regression analysis evaluating the impact of HLA-DR mismatches on the development of anti-DQ dnDSAs, we found no significant association (HR: 1.27; 95% CI: 0.52–3.15, p = 0.6). This suggests that HLA-DR mismatches did not significantly influence the occurrence of anti-DQ dnDSAs in our cohort.

We appreciate your suggestion to include this additional information, which we believe enhances the completeness of our analysis and manuscript.

2. Prior studies have demonstrated that the number of DQ mismatches influences the development of DSAs. Here, the authors have focused on specific allele mismatches and development of DSAs. What was the distribution of single vs double mismatches in for each allele? In other words, were there allele mismatches that were more likely to be found in isolation and allele mismatches that were more likely to be found in a double mismatch? This could also influence differences in development of DSAs.

**Responses:**

Thank you for your insightful comment regarding the distribution of single vs. double MM for each HLA-DQ antigen and their potential influence on DSA development. We performed further analysis to address this question, and the detailed distribution of single and double MM for each specific HLA-DQ antigen has been provided in Supplement Table 1.

We found that the incidence of DSA development was 11.1% in recipients with a single MM and 17.3% in those with double MM (p = 0.124). While double MM appeared to carry a higher risk of DSA formation, likely due to an increased likelihood of antigen-induced immune responses, the rate of DSA development in double MM was not more than twice that of single MM. This suggests that while double MM may increase the risk, their impact on DSA development may not be solely proportional to the number of MM.

To improve clarity, we have updated the relevant statements in the manuscript as follows:

1. “Among them, 75 recipients had 2 HLA-DQB1 MM, bringing the total number of DQB1 mismatches in this study to 566. Details of the distribution of HLA-DQB1 MM are provided in Supplement Table 1.”

2. “The presence of dnDSAs against HLA-DQB1 was detected in 59 patients (12%) at a median of 4.23 years (IQR: 1.97–5.56) after KT. The mean MFI of the first maximum dnDSA was 9,274 ± 6,405. The incidence of HLA-DQ DSA development was 11.1% and 17.3% in patients with single and double MM, respectively.”

3. “The current concept regarding HLA and epitope mismatches focuses on the number of mismatches rather than the specific HLA types (30,31). Our study also revealed that recipients with double DQ MM tended to be at higher risk for DSA development than those with single MM (17.3% vs. 11.1%). However, the rate of DSA development in double MM was not twice as high as in single MM, suggesting that the quality of MM may play a more meaningful role in DSA formation than the quantity alone.”

We hope this additional analysis and the revised manuscript text adequately address your comment and improve the clarity and scientific depth of our study. Thank you again for your valuable suggestion.

3. How was AMR defined?

**Responses:**

Thank you for your comment. The diagnosis of AMR in our study was based on the Banff classification 2019. Pathological scoring, including glomerulitis (g) and peritubular capillaritis (ptc) scores, along with the presence of DSA and C4d staining, was reviewed to make the diagnosis. The revised text now reads:

"Diagnosis of AMR was determined by reviewing pathological scores, DSA results, and C4d staining in accordance with the Banff classification 2019 criteria."

We appreciate your suggestion, which has helped improve the clarity of our manuscript.

4. Was there a specific protocol for induction immunosuppression, or was this left to the discretion of the treating provider? Similarly, can you provide additional information on how maintenance immunosuppression was decided?

**Responses:**

Thank you for your comment regarding our induction and maintenance immunosuppression protocols. At our center, we follow a specific protocol for induction immunosuppression. IL-2 receptor antagonists are used for non-HLA-identical kidney transplant recipients, while antithymocyte globulin (ATG) is administered to high-risk recipients, such as those with high panel reactive antibody (PRA) levels. However, prior to 2012, most patients did not receive antibody induction due to limitations in reimbursement.

For maintenance immunosuppression, the initial regimen generally consisted of tacrolimus, mycophenolate mofetil (MPA), and prednisolone. In some cases, changes in immunosuppressive therapy were necessary due to clinical circumstances. For example, MPA was replaced with mTOR inhibitors in patients with BK virus viremia/viruria, persistent CMV viremia, mycobacterial infections, or those who experienced adverse effects from MPA. Similarly, tacrolimus was switched to mTOR inhibitors in patients with calcineurin inhibitor (CNI) nephrotoxicity or cancer. Final decisions regarding changes in immunosuppressive regimens were made at the discretion of the treating physician, based on individual patient needs.

To improve clarity, we have revised the text in the manuscript as follows:

"Depending on the immunologic risk, induction with basiliximab or antithymocyte globulin was prescribed. However, prior to 2012, most patients did not receive antibody induction due to reimbursement limitations. The initial immunosuppressive regimen at our center consisted of mycophenolic acid, prednisolone, and either cyclosporine or tacrolimus during the earlier period, with tacrolimus becoming the standard during the later phase. Calcineurin inhibitors (CNI) or mycophenolate mofetil were substituted with mTOR inhibitors in cases of infections (e.g., BK viruria/viremia, persistent CMV viremia, or mycobacterial infections), CNI nephrotoxicity, or malignancy. Changes to immunosuppressive regimens were made based on the clinical judgment of the treating physician."

5. The tables, their abbreviations/notes and the figure legends are embedded in the main text and this makes following the text more difficult. I would recommend placing the tables and figure legends at the end of the manuscript.

**Responses:**

Thank you for your valuable suggestion. We agree that placing the tables, their abbreviations/notes, and the figure legends at the end of the manuscript will improve readability and make the text easier to follow. We have made the necessary adjustments by moving all tables, abbreviations/notes, and figure legends to the end of the manuscript, as recommended.

We appreciate your feedback, which has enhanced the clarity and organization of our manuscript.

6. In the methods you mention that "Simultaneous kidney-pancreas patients should be removed from the analysis, given the different potential for Ab development and rejection" - if SPK patients were indeed included, I would recommend removing them from the analysis

**Responses:**

Thank you for your observation and suggestion. We would like to clarify that SPKT recipients were not included in our study. For better clarity, we have revised the manuscript to explicitly state this exclusion criterion. The updated text now reads:

"A retrospective cohort study was conducted among adult patients who underwent KT at our center between January 2006 and December 2020. We included recipients with at least one HLA-DQB1 MM. Patients were excluded if they underwent repeat KT, combined transplantation, had pretransplant donor-specific antibodies, experienced primary allograft failure, or lacked post-transplantation data on HLA antibody results."

We hope this revision resolves any ambiguity and improves the clarity of our methodology. Thank you for your valuable feedback.

7. In the introduction, it is stated that "compatibility of HLA between donors and recipients is crucial in achieving successful KT" - I would revise this sentence, as many recipients achieve excellent outcomes despite receiving fully mismatched kidneys.

**Responses:**

Thank you for your insightful comment. We agree that excellent outcomes can often be achieved in kidney transplantation (KT) even with fully mismatched kidneys. However, clinical evidence supports that greater HLA compatibility between donors and recipients is associated with improved outcomes. To address your comment and ensure accuracy, we have revised the statement in the manuscript to reflect this nuance. The updated text now reads:

“Greater compatibility of HLA between donors and recipients is associated with better outcomes in kidney transplantation.”

We hope this revision aligns with your suggestion and provides a more balanced perspective. Thank you for your valuable feedback.

8. Line 199: "The prevalence of dnDSA development per each HLA-DQB1 MM was 11.25% (64/569 MM)." - the number of mismatches should be 566, consistently with the rest of the manuscript.

**Responses:**

Thank you for pointing out this inconsistency in the manuscript. We apologize for the error and have corrected the number of mismatches to 566 for consistency. The revised text now reads:

"The prevalence of dnDSA development per each HLA-DQB1 MM was 11.31% (64/566 MM)."

We appreciate your careful review, which has helped improve the accuracy and clarity of our manuscript.

9. Table 1: "Immunosuppression at time of dnDSA detection" by definition cannot be applied to the "no DSA" column. So what was the time point used to assess the immunosuppression reported?

**Responses:**

Thank you for your insightful comment regarding the immunosuppression data in Table 1. We acknowledge that the label "Immunosuppression at time of dnDSA detection" may have caused confusion. The immunosuppression regimen was determined at the time of dnDSA development in dnDSA-positive patients and at the time of data collection in dnDSA-negative patients. To clarify this, we have revised the wording in Table 1 to:

"Immunosuppression at time of dnDSA detection or at time of analysis."

We appreciate your careful review and valuable suggestion, which has helped improve the clarity and accuracy of our manuscript.

**From Reviewer #3,**

The manuscript describes a single-center, retrospective, cohort study in 491 kidney transplant recipients where they sought to identify the HLA-DQ mismatches with the highest risk of developing de novo DSA. Patients included in this study have at least one HLA-DQ mismatch, have not received a previous kidney transplant, and did not show DSA >1000 MFI pre-transplant. The authors found that 12% developed de novo DSA to HLA-DQ mismatches and multivariate analysis showed that those with DQ7 and DQ9 mismatches carried an increased risk for developing de novo DSA, despite not being the most found DQ mismatches. Finally, they show that patients who developed de novo DQ DSA, compared to patients without de novo DQ DSA, had an increased incidence of late active or chronic AMR and lower 10-year death-censored graft survival. This is a well-written paper on a currently popular topic. This manuscript adds to the existing knowledge about HLA-DQ mismatches and de novo DSA formation and its clinical relevance in transplantation.

1. Materials and Methods section, page 6, starting in line 96: Please describe more extensively how the assignment of HLA-DQ DSA was done

1.a. Was DSA assigned at the antigen or allele level (manuscripts state that HLA typing was performed by intermediate or high resolution, depending on whether it was before vs after 2014)

**Responses:**

We thank the reviewer for the comment regarding the level at which DSA were assigned. In our study, DSAs were determined at the antigen level for most HLA-DQB1 mismatches, which is standard practice in solid organ transplantation. An exception was made for specific subsets within the DQB1*03* group: DQB1*03:01, DQB1*03:02, and DQB1*03:03, which we categorized as DQ7, DQ8, and DQ9, respectively.

To clarify this in the manuscript, we have revised the relevant statement as follows:

"The presence of dnDSAs was determined at the antigen level and confirmed using the Luminex LABScreen single antigen method with a mean fluorescence intensity (MFI) cutoff of 1000."

1.b. When you assigned DSA by antigen, how did you handle cases where multiple beads with the same antigen, but different alleles were above your cutoff?

**Responses:**

We appreciate your comment regarding the assignment of DSAs in cases where multiple beads with the same antigen but different alleles were above the MFI cutoff. In our HLA laboratory, any positive bead corresponding to a mismatched antigen was defined as a DSA. For cases with multiple positive beads for the same antigen, the MFI value was assigned based on the bead with the highest MFI.

Additionally, we believe that the significant increase in DSA prevalence observed with HLA-DQ7 and HLA-DQ9 mismatches is robust. These findings are consistent even at the allele level (e.g. DQ7 = DQB1*03:01) and are unlikely to result from overestimation due to multiple alleles counts within the same antigen mismatch.

To improve clarity, we have updated the relevant statement in the manuscript to:

"The presence of dnDSAs was determined at the antigen level and confirmed using the Luminex LABScreen single antigen method with a mean fluorescence intensity (MFI) cutoff of 1000. The maximal MFI was determined from the bead with the highest MFI in cases where multiple beads for the same antigen were positive."

1.c. DQA DSA independent from DQB DSA or DQA-DQB DSA as heterodimer

**Responses:**

We appreciate the reviewer’s comment regarding the distinction between DQA-DSA and DQB-DSA, as well as their potential formation as heterodimers. As outlined in the Methods and Discussion sections, high-resolution HLA typing and DQA information were not available in our center prior to 2014. Therefore, in our analysis, DQA-DSAs were assessed independently from DQB-DSAs to maintain consistency across the entire cohort. It is important to note that the prevalence of isolated DQA-DSA in our population was very low.

As discussed in the manuscript, our population is relatively homogeneous, leading to limited variation in DQA alleles associated with DQB antigens. Furthermore, the detection of dnDSAs currently relies predominantly on HLA antigen-based methods rather than epitope-level assessments. Additionally, routine high-resolution HLA typing is not widely implemented in many developing countries, including most transplant centers in our region, due to the associated costs. As a result, focusing on antigen-level mismatches may be a more practical approach in these settings, particularly in the context of organ allocation and DSA surveillance.

2. Results section,

2.a. Page 8, line 142: Please briefly describe how the virtual XM was done.

**Responses:**

Thank you for your comment requesting clarification on the virtual crossmatch process. In our center, patients with high panel reactive antibody (PRA) levels typically underwent single antigen bead testing. The results from this testing were then compared with donor HLA typing. If no preformed donor-specific antibodies (DSAs) were detected in the comparison, the kidney was accepted for transplantation.

We have revised the manuscript to provide a more detailed description. The updated text now reads:

"All KT had negative complement-dependent cytotoxicity (CDC) and antihuman globulin (AHG) crossmatches. Virtual crossmatch was also performed in patients with high PRA levels, most of whom had single antigen bead testing results."

We hope this clarification addresses your concern and improves the clarity of our methodology.

2.b. Page 12

2.b.i. Line 197: “the most common specific anti-DQ dnDSA was against HLA-DQ7 (24%)”, however, figure 2A shows 23.44% which rounded to the nearest whole number is 23%. Please clarify.

**Responses:**

We thank the reviewer for pointing out this discrepancy. We acknowledge this error and apologize for the oversight. The correct percentage, as shown in Figure 2A, is 23.44%, which rounds to 23%. We have corrected this in the revised manuscript to ensure consistency between the text and the figure.

2.b.ii. Line 198: 18% for DQ9 but figure 2A shows 17.19% which rounded to the nearest whole number is 17%. Please clarify.

**Responses:**

We thank the reviewer for pointing out this discrepancy. We acknowledge this error and apologize for the oversight. The correct percentage, as shown in Figure 2A, is 17.19%, which rounds to 17%. We have corrected this in the revised manuscript to ensure consistency between the text and the figure.

2.b.iii. Line 200: The prevalence of DQ dnDSA per DQ antigen says DQ9 is 16.92% but figure 2B shows 16.67%, DQ2 says 12.85% but the figure shows 14.08%. Please clarify.

**Responses:**

We sincerely thank the reviewer for identifying this discrepancy. Upon re-analyzing the data, we found an error in the original analysis. The corrected prevalence of anti-DQ dnDSA per mismatch is now accurately reported as 16.92% for DQ9 and 14.28% for DQ2. We have updated the corresponding text in the Results section to reflect this correction:

"The highest prevalence of anti-DQ dnDSA per MM was found in patients who had HLA-DQ7 MM (17.44%), followed by HLA-DQ9 (16.92%) and HLA-DQ2 MMs (14.28%). In contrast, the lowest prevalence was revealed in patients who had HLA-DQ6 MM (5.43%).”

We have also replaced Figure 2 with the revised version to ensure consistency between the figure and the text. We apologize for this oversight and appreciate the reviewer’s careful attention to detail, which has helped improve the accuracy and clarity of our manuscript.

2.c.i. Line 215-220 is confusing. Why are DQ7 mismatches compared to DQ5 and DQ6, and DQ9 compared to DQ6?

**Responses:**

Thank you for your valuable comment. We agree that the description in lines 215–220 may have been confusing. The Kaplan–Meier analysis compared the risk of dnDSA occurrence across various HLA-DQ mismatches. However, statistically significant differences were observed only in the comparisons of DQ7 vs DQ5, DQ7 vs DQ6, and DQ9 vs DQ6. No significant differences were found in other DQ antigen comparisons. Therefore, we focused on reporting these significant findings in the manuscript.

To clarify, we have revised the text as follows:

"Kaplan–Meier analysis revealed that recipients carrying HLA-DQ7 MM had a higher chance of dnDSA occurrence compared to those with HLA-DQ5 (log-rank p = 0.017) and HLA-DQ6 (log-rank p = 0.005), respectively. Recipients with HLA-DQ9 MM faced a significantly higher risk of dnDSA development than those with HLA-DQ6 (log-rank p = 0.026). No statistically significant differences were observed in other DQ antigen comparisons (Fig. 3)."

We believe this adjustment improves clarity and ensures that the text accurately reflects the results of the Kaplan–Meier analysis.

2.c.ii. Line 226-228, like 4.a., why are DQ7 and DQ9 compared to DQ6?

**Responses:**

Thank you for your thoughtful comment. We agree that the rationale for comparing DQ7 and DQ9 mismatches to DQ6 may not have been sufficiently explained. Our adjusted Cox model compared the risk of dnDSA development across different HLA-DQ mismatches. However, statistically significant differences were observed only for DQ7 vs DQ6 and DQ9 vs DQ6. No other comparisons yielded statistically significant differences. Therefore, we reported only the significant findings in the manuscript to focus on the key results.

To improve clarity and avoid confusion, we have revised the text as follows:

" The adjusted Cox model demonstrated that recipients with HLA-DQ7 MM had the highest risk of dnDSA formation (HR: 2.80; 95% CI: 1.21 – 6.52; p = 0.017), followed independently by those with HLA-DQ9 MM (HR: 2.63; 95% CI: 1.11 – 6.27; p = 0.028) (Table 2). When specifically compared to recipients with HLA-DQ6 MM, those with HLA-DQ7 MM (HR: 3.67; 95% CI: 1.20 – 11.25; p = 0.023) and HLA-DQ9 MM (HR: 3.391; 95% CI: 1.08 – 10.65; p = 0.037) had a significantly higher risk of dnDSA occurrence. No statistically significant differences were observed in other pairwise comparisons of DQ MM."

We believe this revision improves the clarity of our findings and provides a more accurate interpretation of the statistical results.

2.c.iii. Line 234 and figure 4A shows that patients with dnDSA to DQ have lower rejection-free survival compared to patients with no dnDSA to DQ.

**Responses:**

Thank you for your comment regarding the interpretation of Figure 4A. We believe the text in line 234 is accurate. The Kaplan–Meier analysis in Figure 4A shows that recipients with dnDSAs to HLA-DQ had significantly inferior rejection-free survival compared to those without dnDSAs to HLA-DQ. The reported hazard ratio (HR: 7.76; 95% CI: 5.00–12.03; P < 0.0001) reflects this difference. Therefore, we respectfully suggest that the text aligns with the data presented in the figure.

However, if further clarification is required, we can revise the text to:

"Kaplan–Meier analysis revealed that recipients with dnDSAs to HLA-DQ had significantly inferior rejection-free survival compared to those without dnDSAs to HLA-DQ (HR: 7.76; 95% CI: 5.00–12.03; P < 0.0001; Fig 4A)."

We hope this clarification resolves any confusion and accurately reflects the data presented.

2.c.iii.1. Were there any differences between different DQ DSA (i.e., DQ7 or DQ9 DSA worse than other DQ DSA?

**Responses:**

Thank you for your insightful comment regarding potential differences in outcomes between specific HLA-DQ DSAs, such as DQ7 or DQ9. We agree that this is a critical and interesting area of investigation. Our center is currently conducting both retrospective and prospective studies to further evaluate the long-term outcomes, including the severity of rejection pathology associated with different DSA types, particularly focusing on specific HLA-DQ antibodies.

We recognize the importance of this question, and the findings from these ongoing studies will help to elucidate this issue. We look forward to reporting these results in future publications.

2.c.iii.2. How about DQ DSA versus non-DQ DSA? In other words, is it clinically relevant that certain DQ mismatches seem to be more “immunogenic”?

**Responses:**

We sincerely thank the reviewer for raising this insightful question regarding the clinical relevance of different DSAs and the potential immunogenicity of specific DQ mismatches. Your comment prompted us to perform additional analyses, which revealed several important findings.

We found that 20 patients (4.1%) had isolated non-DQB1 DSAs without corresponding DQB1 DSAs. Kaplan-Meier analysis showed no significant difference in rejection-free survival between recipients with HLA-DQ dnDSAs and those with non-DQ DSAs (log-rank P = 0.111). However, recipients with dnDSAs to HLA-DQ had significantly lower overall graft survival compared to those with non-DQ DSAs (log-rank P = 0.038). Additionally, no significant difference in graft survival was observed between recipients with non-DQ DSAs and those without DSAs (log-rank P = 0.67).

These findings suggest that anti-DQ DSAs may exert a more detrimental impact on long-term graft outcomes compared to non-DQ DSAs. We have incorporated these results into the revised manuscript, along with three new supplemental figures. We greatly appreciate your valuable suggestion, which has added further depth and clarity to our study.

3. Discussion section

3.a. Page 16, line 295: When the authors refer to ratio of dnDSA per MM, shouldn’t this use the rank and percentages on figure 2B rather than 2A?

**Responses:**

Thank you for your valuable observation. We agree that the ratio of dnDSA per mismatch should be based on the percentages provided in Figure 2B rather than Figure 2A. We acknowledge this error and have corrected the statement in the manuscript. The revised text now reads:

"Therefore, when analyzing the ratio of dnDSA per MM, the most frequently occurring specific DQ antibodies were against DQ7 (17.4%), followed by DQ9 (16.9%) and DQ2 (14.3%)."

We appreciate your attention to this detail, which has helped improve the accuracy of our manuscript.

3.b. Page 16, line 303, the study cited is from Canada and not from the US

3.c. Page 7, line 313, the study is from Canada and not from the US

**Responses:**

Thank you for your valuable observation. We acknowledge the error regarding the country of origin for the cited study and have corrected the statements in the manuscript. The revised text now reads:

“A study on heart and/or lung transplant recipients from Canada showed that an epitope mismatch of HLA-DQA105+DQB102 or DQB107 increased the risk for dnDSA occurrence by approximately fourfold.”

“Our findings, in line with previous Canadian data in heart and lung transplantation (8), support the notion that the problematic mismatch might be DQ7 more than the colocalized DQA.”

We appreciate your attention to detail, which has helped improve the accuracy and clarity of our manuscript.

Finally, we would like to express our gratitude for the valuable suggestions provided by the reviewers and editors, which have significantly enhanced the quality of our manuscript. We greatly appreciate the helpful comments and thank you for dedicating your time to review our study. The manuscript has benefited substantially from the recommended revisions.
